# Perineural local anaesthetic catheter after major lower limb amputation trial (PLACEMENT): study protocol for a randomised controlled pilot study

**DOI:** 10.1186/s13063-017-2357-x

**Published:** 2017-12-28

**Authors:** David C. Bosanquet, Graeme K. Ambler, Cherry-Ann Waldron, Emma Thomas-Jones, Lucy Brookes-Howell, Mark Kelson, Tim Pickles, Debbie Harris, Deborah Fitzsimmons, Neeraj Saxena, Christopher P. Twine

**Affiliations:** 1 Aneurin Bevan University Health Board, Royal Gwent Hospital, Cardiff Road, Newport, NP16 2UB UK; 2 Division of Population Medicine, Cardiff University, 5th Floor, Neuadd Meirionnydd, Heath Park, Cardiff, CF14 4XW UK; 3 Centre for Trials Research, Cardiff University, 7th Floor, Neuadd Meirionnydd, Heath Park, Cardiff, CF14 4XW UK; 4 Swansea Centre for Health Economics, College of Human Health Sciences, Swansea University, Singleton Park, Swansea, SA2 8PP UK; 5 Department of Anaesthetics, Royal Glamorgan Hospital, Cwm Taf Local Health Board, Llantrisant, UK; 6 School of Psychology, Cardiff University, Cardiff, CF10 3AX UK; 7 Psychology and Therapeutic Studies, University of South Wales, Pontypridd, CF37 1DL UK

**Keywords:** Amputation, Vascular Surgery, Peripheral Arterial Disease, Perineural Catheter, Randomised Controlled Trial, Pain, Feasibility, Pilot

## Abstract

**Background:**

Pain after major lower limb amputation for peripheral arterial disease (PAD) is a significant problem. A perineural catheter (PNC) can be placed adjacent to the major nerve at the time of amputation with a continuous local anaesthetic infusion given postoperatively to try and reduce pain. Although low-quality observational data suggest that PNC usage reduces postoperative opioid requirements, there are limited data regarding its effect on pain. The aim of PLACEMENT is to explore the feasibility of running an effectiveness trial to assess the impact of a PNC with continuous local anaesthetic infusion, inserted at the time of amputation, on short and medium-term postoperative outcomes.

**Methods/design:**

Fifty patients undergoing a major lower limb amputation (below or above the knee) for PAD will be recruited from two centres. Patients will be randomised in a 1:1 ratio to receive standard postoperative analgesia, with or without insertion of a PNC and local anaesthetic infusion for the first 5 postoperative days. Outcome data will be captured for the first 5 days, including pain scores (primary outcome, captured three times a day), opioid use, nausea or vomiting, itching, dizziness and complications. Patients will be contacted 2 and 6 months after surgery to assess quality of life, phantom limb pain, chronic stump pain and total healthcare costs. Semi-structured interviews will be conducted with at least 10 patients (dependent on saturation of analytic themes on preliminary coding) purposefully sampled to achieve variation in site and study arm. Interviews will explore patients’ perception of post-amputation pain and its treatment, and experience of study processes. Semi-structured interviews with 5–10 health professionals will explore feasibility, fidelity, and acceptability of the study. Data from this pilot will be used to assess feasibility of, and estimate parameters to calculate the sample size for an effectiveness trial. Full ethical approval has been granted (Wales Research Ethics Committee 3 reference number 16/WA/0353).

**Discussion:**

PLACEMENT will be the first study to explore the feasibility of running an effectiveness trial on PNC usage for postoperative pain in amputees, and provide parameters to calculate the appropriate sample size for this study.

**Trial registration:**

ISRCTN.com, ISRCTN85710690. Registered on 21 October 2016.

European Clinical Trials Database (EudraCT), 2016-003544-37. Registered on 24 August 2016.

**Electronic supplementary material:**

The online version of this article (doi:10.1186/s13063-017-2357-x) contains supplementary material, which is available to authorized users.

## Background

Peripheral arterial disease (PAD) affects approximately 20% of people aged over 55 years, of whom 1–2% will eventually require a lower limb amputation [1, 2]. The management of postoperative pain in this population is essential for these patients’ short-term recovery and ongoing rehabilitation. Amputees suffer with different types of pain: immediate postoperative pain, chronic stump pain (CSP) and phantom limb pain (PLP). Despite numerous studies, determining how to best manage such patients to reduce both acute and chronic pain is not clear [3].

Immediate postoperative pain management commonly involves epidural or intravenous patient controlled analgesia, reliant upon opioid-based agents. The recent UK-wide National Confidential Enquiry into Patient Outcome and Deaths (NCEPOD) review into lower limb amputations found that strong opioids were the most frequently used type of analgesia postoperatively [4]. Whilst providing excellent pain relief, opioids have many side effects including nausea, vomiting [5], constipation [6], sedation and pruritis [7]. Tolerance, dependence and addiction can occur with prolonged use. Opioid-related side effects are problematic; they increase in-hospital costs by 7–16% and length of stay by around 10% [8, 9].

Given the marked side effects of opioids, the American Society of Anesthesiologists suggests using multimodal analgesic strategies for managing postoperative pain [10], which includes utilising peripheral wound catheters (PWCs) with local anaesthetic infusion where possible. PWCs are thin catheters placed within the surgical wound. Perineural catheters (PNCs) are a specific type of PWC placed adjacent to major nerves, which allow a continuous infusion of local anaesthetic directly to the perineural (juxta-neural) space [11]. PWCs can be placed under direct vision at the time of surgery by the operating surgeon (surgical PWCs), or percutaneously under radiological guidance either preoperatively or postoperatively (radiological PWCs). In large meta-analyses, PWC usage is associated with reduced postoperative pain scores at rest and on movement, opioid usage, nausea and vomiting, length of hospital stay and increased patient satisfaction [12], with minimal risk [13, 14].

Use of PNCs for major amputations, inserted adjacent to either the sciatic nerve (for above-knee amputations (AKAs)) or the tibial nerve (for below-knee amputations (BKAs)), was first described in 1991 [15]. A recent systematic review of PNC use in lower limb amputation identified seven reports of studies comprising 416 patients [16]. Two were prospective randomised controlled trials (RCTs) [17, 18] whilst the remainder were observational studies. On meta-analysis, opioid use was less with the PNC compared to controls (standardised mean difference -0.59, 95% confidence interval (CI) -1.10 to -0.07; *p* = 0.03) [16]. Only two papers provided data on postoperative pain, with no difference being demonstrated, although numbers of patients included were low and comparison groups were non-standardised. There was no effect on mortality, CSP or PLP. Importantly, no trials had adequate power to detect a difference in postoperative pain. A number of other papers not included in the meta-analysis have suggested that PNCs reduce postoperative pain [19–22].

Pain after amputation is a significant problem for patients. The recent 2014 NCEPOD report of lower limb amputations suggested only 37.5% of patients receive “good” pain control postoperatively. Postoperative pain has been shown to be one of the vital factors in patients’ overall satisfaction with their operative experience [23]. The use of a PNC may reduce both postoperative pain and opioid use, and therefore also opioid side effects such as nausea, vomiting, constipation and delirium. In the absence of robust data, a contemporaneous RCT is necessary to confirm or refute its value in amputees, in terms of pain relief, opioid use, quality of life (QoL) and overall cost. The PLACEMENT study aims to recruit 50 patients undergoing major lower limb amputation for PAD, and assess the feasibility of randomising them in a 1:1 ratio to receive a PNC with a continuous local anaesthetic infusion for up to 5 days, inserted at the time of amputation, or not. Both groups will receive personalised anaesthetic and postoperative analgesic regimes. While the primary objective is the assessment of feasibility, it is also necessary to define effectiveness outcomes, as the unexpected detection of a significant deleterious effect would be a strong reason not to proceed with an effectiveness trial. The primary effectiveness outcome will be short-term pain, with secondary effectiveness outcomes including opioid use, opioid side effects, long-term pain, QoL and a cost analysis. A full effectiveness trial will be deemed appropriate following careful evaluation of the pilot study against published criteria, if at least 25% of eligible patients are enrolled, and as long as a minimum of 60% of patients provide primary outcome data. This protocol has been developed in line with the Standard Protocol Items: Recommendations for Interventional Trials (SPIRIT) 2013 statement [24] Additional file 1.

## Methods/design

### Objectives

The primary objective of PLACEMENT is to assess the feasibility of running an effectiveness trial to determine whether the use of a PNC with local anaesthetic infusion reduces pain in the first 5 postoperative days in amputees. Key parameters permitting progression to an effectiveness trial include effective study design and management, according to the Acceptance Checklist for Clinical Effectiveness Pilot Trials (ACCEPT) criteria [25], described in detail later, the Trial Steering Committee (TSC) and Trial Management Group (TMG) must approve the study as safe, without excessive complications in either study arm, the percentage of eligible patients that are randomised must be greater than 25%, and the percentage of randomised patients that provide data for the primary outcome must be greater than 60%.

Secondary objectives include: to estimate an effect size (to allow the appropriate sample size to be calculated for a future RCT); identify barriers to patient recruitment and site set up; identify the proportion of eligible participants who consent to the study; identify the proportion of consented participants who supply primary outcome data; identify what frequency of pain score measurements, and using which tool, are most suited to identify pain in this cohort of patients; identify the proportion of patients reaching follow up to evaluate PLP and CSP levels; identify which secondary outcomes are important to include, and how best to assess them; develop a greater understanding of overall patient experience relating to pain, and pain management, following a major amputation; explore the feasibility of blinding the surgical and anaesthetic teams to study allocation preoperatively; and develop a suitable framework for a full health economic evaluation in a future RCT.

In parallel with this work, we are also undertaking research to identify the most important outcomes (core outcome sets) for future amputation research. A protocol for this work is reported separately [26].

### Design

PLACEMENT is a pragmatic randomised non-blinded 1:1 pilot study. In the UK, it is classified as a clinical trial of an investigational medical product (CTIMP) type A, as both arms use procedures and medications that are already used in routine clinical practice.

### Participants

Fifty participants will be enrolled over 12 months. Adults undergoing major lower limb amputation, either BKA (trans-tibial) or AKA (trans-femoral), for complications of PAD are considered. Patients with PAD were considered the most suitable for this study as in the UK PAD is the most common reason for a patient requiring an amputation [27], and other less common indications, particularly trauma, are associated with extremes of phantom limb pain [28]. The pharmacokinetics of opioids are altered with increasing age, deteriorating renal function and polypharmacy, attributes commonly encountered in patients with PAD but far less common in patients having amputations for other reasons, leading to unpredictable adverse drug effects and interactions [29]. Finally, the NCEPOD can provide clear data on recent UK amputation practice [4], providing motivation for this study.

### Inclusion criteria

The following patients will be suitable for inclusion in the study: patients aged 18 years or older, undergoing elective or emergency major lower limb amputation (BKA, or AKA) for complications of PAD, able to assess pain using a verbal rating scale (VRS) and with a life expectancy longer than 2 weeks. For women of childbearing potential, they need to be willing to undergo a pregnancy test before the study and agree to either use a highly effective method of contraception or abstain from sexual intercourse until at least 7 days after their amputation.

### Exclusion criteria

The following patients will be excluded from enrolling in the study: patients undergoing digital, metatarsal or tarsal amputation, disarticulation of the hip or hindquarter amputation, simultaneous bilateral amputations or through-knee amputation. Patients who are unable to provide consent due to incapacity (as defined by the Mental Capacity Act 2005) will be excluded, as will vulnerable or protected adults, and pregnant females. Patients with an allergy or intolerance to any of the substances in the PNC, or local anaesthetic agents, or chronically taking class IB anti-arrhythmic agents or local anaesthetic agents, for example in the form of transdermal patches, will be excluded, as will patients expected to be managed in the intensive care unit postoperatively and be sedated for more than 24 hours. Patients undergoing a subsequent amputation who have already been enrolled to participate in the PLACEMENT study will not be recruited again.

Patients who meet the inclusion criteria but who subsequently are not suitable include patients in whom a planned major amputation was not performed (due to either anaesthetic or surgical difficulties), patients in whom a major amputation was performed but the appropriate nerve was not identified, patients who, due to instability in the intra-operative period, require admission to the intensive care unit postoperatively and are most likely to be sedated for more than 24 hours, and patients who, due to instability in the intra-operative period, are not expected to survive more than 2 weeks.

### Trial interventions

#### Intervention arm

Participants randomised to the active treatment arm receive usual care with levobupivacaine hydrochloride 0.125% delivered to the perineural space via a PNC placed at the time of the amputation, at a dose of 2.5–15 mg/h, continued for up to 5 days. All participating surgeons will be assessed and trained prior to study enrolment to ensure PNC placement is standardised across the two sites. Local anaesthetic infusion will be via an elastomeric pump, with standard monitoring of the pump during the period of its use.

For PNC placement, the sciatic nerve (for AKA) or tibial nerve (for BKA) is identified. The nerve is transected sharply under gentle tension, to allow the cut end of the nerve to retract from the wound to reduce neuroma formation [30]. An epidural catheter (20G, 0.85 mm diameter) is used as the PNC, and placed in the perineural space under direct vision after the limb has been removed. A non-coring Tuohy needle is first placed in the perineural space, the fenestrated end of the epidural catheter advanced along the needle so to lie adjacent to the nerve, approximately 10–20 cm cranial from the cut end, and the needle withdrawn leaving the catheter in place. A suitable exit site for the catheter on the lateral aspect of the amputation stump is selected, and the Tuohy needle passed through the skin (external to internal) at this point. The free end of the epidural catheter is then passed through the needle, which is then withdrawn to leave the catheter exiting the wound. The amputation is completed and the epidural catheter is coiled and secured under an adhesive dressing. No operative blockade of the femoral nerve (for AKA) or peroneal nerve (for BKA) will be performed routinely.

#### Control arm

Participants randomised to the control arm receive usual care (standard anaesthetic and postoperative analgesia alone). The primary mode of anaesthetic (general, epidural, spinal, regional) will be recorded. Regional nerve blocks or PNCs, placed either during the anaesthetic phase or preoperatively, are permitted in either arm. Their use will be recorded, and if a PNC is placed, the duration of postoperative local aesthetic infusion will be recorded. In the instance of a preoperative PNC and a study PNC being placed in the same patient, the local anaesthetic infusion rate will be carefully checked to ensure local anaesthetic toxicity will not occur.

Postoperative analgesia will be determined by the anaesthetic and surgical team, tailored to the patients’ individual needs. As standard, all patients will receive regular paracetamol. Opioids are also prescribed as required, and are given either intravenously via a patient-controlled analgesia device, or orally by the ward nurses. Gabapentinoids or tricyclic antidepressants are used for neuropathic or phantom limb pain. Other analgesic adjuncts such as ketamine infusions are also permitted. Use of all analgesics will be recorded in both intervention and control arms.

### Outcomes

While this is primarily a pilot study, and as such the feasibility objectives listed above are the primary measures of importance, we feel it valuable to also define putative effectiveness outcomes. Depending upon the study results these may, or may not, carry over into a future effectiveness trial.

#### Primary effectiveness outcome

The primary outcome is pain experienced over the first 5 postoperative days, as assessed using a VRS, captured three times a day.

#### Secondary effectiveness outcomes

The following will be captured as secondary outcomes: pain control assessed by the Overall Benefit of Analgesia Score (OBAS) [31] preoperatively and once daily postoperatively for 5 days; pain assessed by the Self-completed Leeds Assessment of Neuropathic Symptoms and Signs (S-LANSS) [32] preoperatively and on postoperative day 5; and opioid use measured preoperatively and once daily postoperatively for 5 days, converted to morphine equivalents. Pain, as assessed by the S-LANSS and the Groningen PLP questionnaire, will be captured at 6-week and 6- month follow up. QoL will be assessed preoperatively and at 2-month and 6-month follow up using the Euroquol-5D-5 L health questionnaire (EQ-5D-5 L) and Hospital Anxiety and Depression Scale (HADS). Surgical-site infection rates, classified as per the 2008 Centers for Disease Control/National Healthcare Safety Network (CDC/NHSN) document, will be recorded. We will also capture the rate of successful identification of the nerve and PNC placement, and resource usage during the first 6 postoperative months.

### Study procedures

#### Site selection and training

This study will be carried out at two tertiary vascular surgery hospital sites in Wales; the Royal Gwent Hospital, Aneurin Bevan University Health Board and Morriston Hospital, Abertawe Bro Morgannwg University Health Board. Full contact details of the recruitment centres can be found in the ISRCTN registration.

#### Registration and consent

**Fig. 1:**
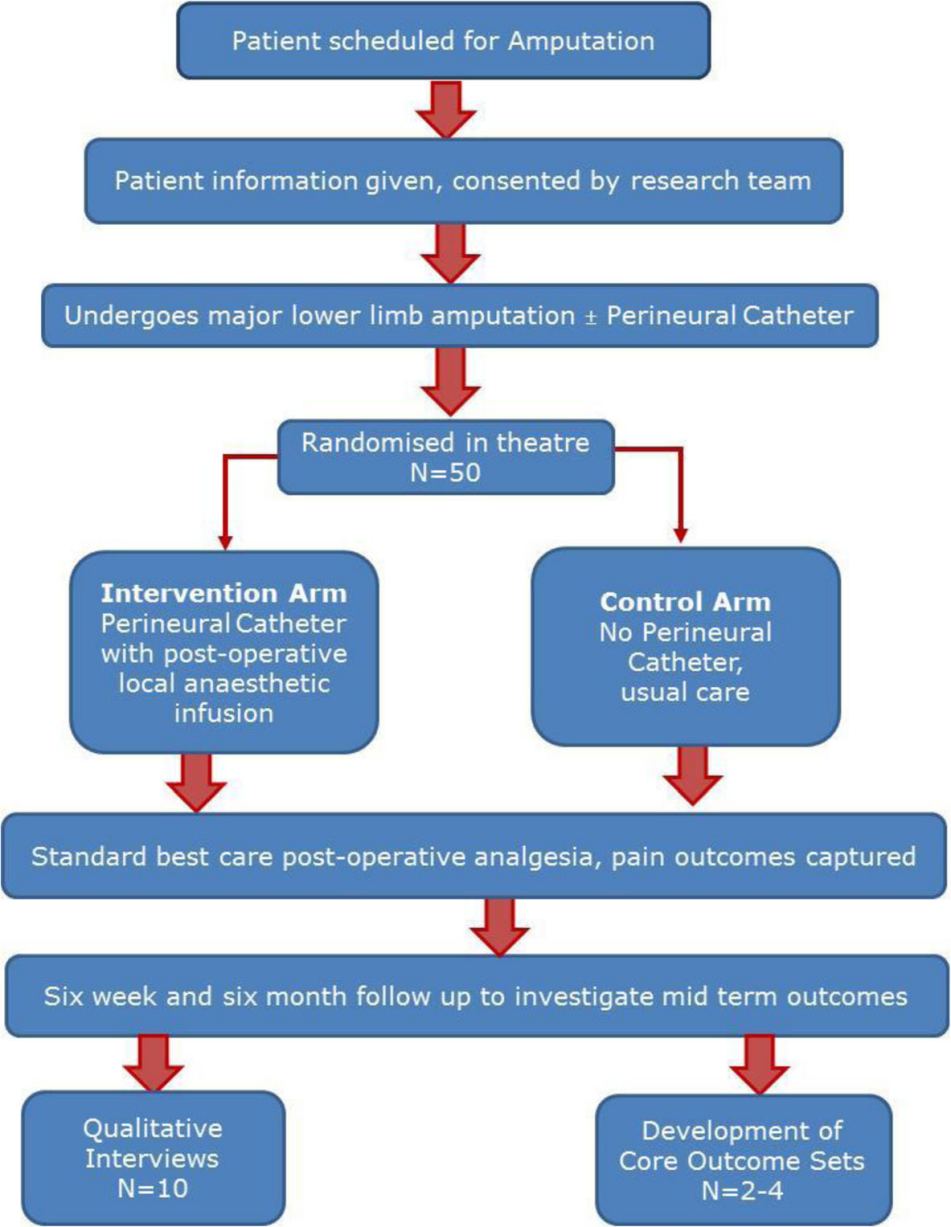
Trial schema and participant flow diagram

The study plan and patient flow are shown in Fig. 1. Patients listed for major lower limb amputation will be screened by members of the clinical care team against the inclusion/exclusion criteria. Potentially suitable patients will be approached, provided with all necessary information Additional file 2, and if appropriate will be consented by a medically trained member of the study team Additional file 3. Patients undergoing emergency surgery will only be approached if their clinical condition allows sufficient time to obtain adequate informed consent. An anonymous screening log of all patients undergoing major lower limb amputation but not enrolled will be maintained at both sites to measure potential selection bias.

### Randomisation

Patients will be randomised intra-operatively (preferably) or preoperatively (if logistically required). Patients will be randomised in a 1:1 ratio to either active treatment (placement of a PNC with a postoperative infusion of 0.125% levobupivacaine in addition to standard anaesthetic and postoperative analgesia) or control (standard anaesthetic and postoperative analgesia alone). Computerised web-based remote randomisation (available 24 hours a day) will be used, with telephone backup during working hours. Randomisation will be minimised according to level of amputation (BKA vs. AKA) and sex, and stratified by site. The feasibility of concealing allocation from for the surgical and anaesthetic team up until the point at which the nerve is identified intraoperatively will be explored. This will be done by providing the facility for either intraoperative randomisation, or for randomisation to be performed preoperatively by an individual distinct from the surgical and anaesthetic team, with the allocation left in a sealed envelope and opened when the nerve is identified.

### Criteria for crossover or withdrawal

Removal of the PNC and/or discontinuation of the local anaesthetic infusion in the intervention arm will be performed if the patient experiences an adverse drug reaction or local site reaction or infection. Postoperative placement of a percutaneous (radiologically guided) PNC in the control arm is permitted at the discretion of the treating clinician. Data collection will continue without modification in the case of patient crossover, but the crossover event will be recorded. Participants may withdraw from the study at any point, without giving reasons. If reasons are given, these will be recorded and reported. Permission will be sought from participants who withdraw to allow data that has already been collected to be used in analysis.

### Data collection

**Fig. 2:**
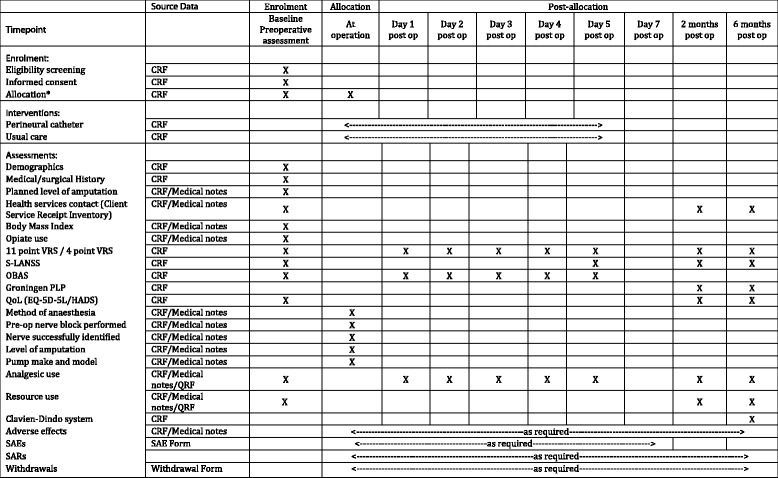
Schedule of enrolment, interventions, and assessments. *Allocation may be done preoperatively or intra-operatively. If allocation is preoperative, attempts will be made to conceal allocation from the surgical and anaesthetic teams up until the point where the nerve is identified, subject to availability of study staff. VRS, verbal rating scale; S-LANSS, Self-completed Leeds Assessment of Neuropathic Symptoms and Signs; OBAS, Overall Benefit of Analgesia Scale; PLP, phantom limb pain; QoL, quality of life; EQ-5D-5 L, EuroQol health questionnaire; SAE, serious adverse event; SAR, serious adverse reaction; CRF, case report form

Preoperative and postoperative data will capture pain, opioid use, nausea and vomiting, and other key measures. Follow-up assessments will be carried out either by telephone or in person at approximately 2 and 6 months. The schedule of enrolment, interventions and assessments is shown in line with the SPIRIT 2013 recommendations in Fig. 2 [24]. Participants completing follow-up assessments will be given incentive vouchers to encourage them to engage with follow up. Pain (both PLP and CSP), analgesic use, complications, QoL and healthcare costs will be assessed. Data will be coded, stored and analysed at the Centre for Trials Research. It will be recorded in a web-based database, which is a secure encrypted system accessed by an institutional password, and complies with UK Data Protection Act standards. The primary outcome will be recorded using the well-validated 11-point (0–10) VRS when captured by study personnel. However, it became clear during the design of the study that it would be difficult to record this more than once each day, as research-trained personnel were not regularly available first thing in the morning, in the evening, or at the weekend. Daily pain scores will therefore also be supplemented by the simpler 0–3 scale (none, mild, moderate, severe), which is recorded routinely by regular nursing staff on the bedside observations chart.

### Qualitative interviews and core outcome sets

In tandem with this research we are undertaking complimentary qualitative work. Semi-structured, face-to-face interviews will be conducted with patients purposefully sampled to achieve variation in centre and study arm. The target sample size is 10, dependent on saturation of analytic themes on preliminary coding and discussion within the research team. The topic guide will be developed in discussion with the multi-disciplinary study team, including lay members, and reviewed and refined throughout the interview process where necessary Additional files 4 and 5. Patients will be interviewed within the immediate postoperative period (expected to be 1–4 weeks after amputation) and again at further follow up (6–9 months after amputation). Interview 1 will explore perceptions of pain including acute postoperative pain and chronic stump pain, general illness experience and the hospital stay, personal experiences of opioid use, and any other concerns. Interview 2 will explore general illness experience including physical and psychological, experience of long term pain (including PLP), current pain relief, recovery, and rehabilitation. Interviews will also explore patients’ experiences of study processes. Interviews will be audio-recorded, transcribed verbatim, and anonymised. The data will be managed using qualitative coding software (NVivo 11). Thematic analysis will be used to identify, analyse and report patterns within the data [33]. Themes will be identified that relate to research objectives, but analysis will also allow new, unpredicted themes to be identified that interviewees generate themselves. Data will be double-coded (around 10–20%) and discrepancies discussed until consensus is reached and codes will be refined if necessary.

Semi-structured interviews (telephone or face-to-face) will be conducted with a total of 5–10 health professionals sampled from both sites involved in delivery of the study Additional files 6 and 7. Interviews will explore how delivery of the intervention was achieved and what was delivered, how the intervention components and delivery processes worked in the real healthcare setting, and acceptability of the study to patients and intervention deliverers. We also plan the parallel development of core outcome sets for amputation research, which is described in detail elsewhere [26]. Patients who agree to participate in these ancillary studies will be given additional information about them and asked to sign additional consent forms.

## Analysis

### Sample size

This study is focussed on feasibility and is not powered to analyse effectiveness. A sample size of 50 participants has been chosen to demonstrate this, which will allow for feasibility outcomes to be conservatively estimated with a 95% CI of ± 13.9 percentage points.

### Statistical analysis

Analysis will be undertaken as per the full statistical analysis plan, which will be implemented after data collection is complete, and to which the analyst will be blinded. The following feasibility outcomes will be examined: the proportion of eligible participants who consent to the study; the proportion of patients who supply primary outcome data; and the proportion of patients reaching follow up, to evaluate PLP and CSP.

The primary effectiveness outcome of pain will be analysed using the average (mean) pain rating on the 11-point VRS across the 5 days post-surgery. Simple linear regression will be used to estimate the treatment effect controlling for amputation type and other key demographics (e.g. age, sex, recruiting site, pre-amputation pain score, anaesthetic type, use of percutaneous nerve blocks/catheters) and the results summarised using regression coefficients, 95% CIs and effect sizes. Secondary analysis of the primary outcome will explore the use of the maximum pain score recorded and the area under the curve, and will also include information on total opioid use during the 5 days. If there are missing data issues with the 11-point VRS, defined as less than 90% of patients having at least one 11-point VRS recorded on each of the 5 postoperative days, we will fall back on the 4-point scores, with 11 point scores where available converted to 4-point values using a validated algorithm [34]. The mode of the recorded values will be used and an ordinal regression model used for analysis. Treatment failure, defined as a patient reaching a pain score of 4 or more on the 11-point VRS, (moderate or severe on the 4-point VRS) will also be compared between arms using logistic regression, albeit no formal hypothesis testing will be conducted.

Secondary effectiveness outcomes will be similarly analysed. Continuous outcomes will be explored using linear regression (standard transformations to improve model fit will be used if indicated) and summarised using regression coefficients with 95% CIs. Logistic regression will be used for dichotomous outcomes and summarised using odds ratios with 95% CIs. Secondary outcomes include: the Overall Benefit of Analgesia Scale (OBAS) score, the S-LANSS score at 5-day follow up, QoL (EQ-5D-5 L), surgical infection rates (yes/no), length of stay and 30-day mortality. All effectiveness outcomes will be analysed in as-randomised groups.

Missing data will be addressed by using complete case analysis. If this excludes more than 20% of participants we will employ multiple imputation and report the impact on the treatment effect alongside the complete case analysis.

### Progression criteria

Progression to an RCT will occur after a complete review of all components of the described pilot study against the ACCEPT criteria [25]. This evaluates all components of the trial including trial design, intervention, the consent process, randomisation, blinding, data management and analysis, research governance, and trial management.

The following criteria are required for progression to an RCT. The observed effect must not exclude a clinically significant benefit to patients in the treatment (i.e. PNC) arm and the Trial Steering Committee (TSC) and Trial Management Group (TMG) must approve the study as safe, without excessive complications in either study arm. For two further feasibility criteria, we will employ a “traffic light” system to identify criteria levels that we deem acceptable (green), possibly acceptable with discussion and amendments (amber) and unacceptable (red). The first of these criteria is that the percentage of eligible patients that are randomised must be greater than 50% (green), or 25–50% (amber), with less than 25% recruitment being a red light. Second, the percentage of randomised patients that provide data for the primary outcome must be greater than 90% (green), or 60–90% (amber), with less than 60% primary outcome data being a red light. The reasons for any amber lights will be scrutinised, paying particular attention to rates of those who meet an intra-operative exclusion criterion and who would otherwise have been randomised, and amendments to study procedures will be necessary before a decision to proceed to an effectiveness trial can be made.

We will also carry out a thorough process evaluation to identify and address problems that might undermine the successful delivery of the intervention in a full effectiveness trial [35]. Should we progress to a full RCT, data from this pilot may be analysed in conjunction with data captured in that trial, using a Bayesian approach. The decision will be made after taking advice from the TSC, and will depend primarily on the methodological similarities between the two studies.

### Ethical and governance approval

The full trial protocol was reviewed and approved by Wales Research Ethics Committee (REC) 3, recognised by the United Kingdom Ethics Committee Authority. All hospital sites received Research and Development (R&D) approval from the respective Health Boards in Wales. A notice of no objection to the clinical trial notification was obtained from the Medicines and Healthcare products Regulatory Agency (MHRA). The REC, Sponsor and Health Boards’ R&D departments will be notified of minor protocol revisions. Major protocol revisions will be presented to the REC and the MHRA if appropriate, and once approved, to the Sponsor and Health Boards’ R&D departments before implementation.

### Management and safety

All key processes will be undertaken according to Cardiff University Centre for Trials Research (CTR) Standard Operating Procedures. Regular (monthly) TMG meetings, including patient and public representatives, will occur during the course of the study. Adverse events and protocol deviations will be monitored by the CTR and reported as required to the TSC who will meet regularly to monitor progress. The TSC will act as the Data Safety Monitoring Board. Given the small size and nature of the study, it was decided by the TSC that no formal interim analysis would be performed. No formal stopping criteria have been drafted, although the TSC have the ability to stop the study if there are safety concerns. The study is coordinated by the CTR, who will monitor and audit study procedures. Monitoring will be conducted independently by a qualified member of CTR staff not participating in the day-to-day study activities at the research site.

### Publication and dissemination of results

All publications and presentations relating to the study will be authorised by the TMG and will be in accordance with the study’s publication policy. In addition to the required final report and monograph for the funding body, we will publish the main study results in international open access peer-reviewed journals and present them at national and international scientific meetings. With the assistance of our collaborators and lay representatives we will disseminate the study findings to a wide audience and vigorously promote uptake of the study results into clinical care. This will include presentations at meetings and written executive summaries for key stakeholder groups such as Secondary Care Trusts, Royal Colleges, Medical Schools, and relevant patient groups. Access to the full protocol, anonymised participant-level data, and statistical code will be available from the study team upon request after the main study results have been published.

## Discussion

PLACEMENT is designed to explore the feasibility of running an effectiveness trial to assess the impact of a perineural catheter (PNC) with continuous local anaesthetic infusion, inserted at the time of amputation, on immediate and mid-term postoperative outcomes. If feasible, an effectiveness trial will address the important evidence gap in postoperative pain relief for amputees. This is an important research priority, highlighted by the recent NCEPOD report [4].

The primary effectiveness outcome was chosen following input from a patient-engagement event with prior amputees, organised during study set-up. The individuals present felt that acute pain in the postoperative period was an important issue. The measures chosen (an 11-point VRS with a back-up of the simpler 4-point VRS recorded on the routine nursing observations chart) reflect those recommended by consensus documents and expert opinion [34, 36].

During the development of this study we decided against a placebo control arm (i.e. insertion of a PNC with an infusion of 0.9% saline), despite the potential for introducing bias, for a number of reasons. First, we wanted to design a pragmatic study that would compare two “real life” treatments, which would be easily applicable to surgeons’ daily practice. Second, the placement of a PNC comes with a theoretical risk of damaging the nerve, which could in theory increase pain. Whilst there are no reports of this occurring within the published literature as identified in our systematic review, we felt that we needed to show whether the placement of a PNC was detrimental. Third, it is possible that infusion of any inert fluid, such as 0.9% saline, would dilute inflammatory mediators in the vicinity of the nerve and thereby reduce pain, potentially resulting in the placebo group experiencing pain relief despite no “active” infusion. Finally, PNC placement may theoretically be associated with an increase in surgical-site infection or other complications, which would not be identified in a placebo study. As the main focus for the present study was feasibility, we decided against the need for a placebo arm. If the feasibility outcomes of the study are achieved, this topic will be re-visited in the planning phase of an effectiveness trial.

During the design of this study we identified two similar studies of note. The first, currently unreported, has been undertaken in Leicester, recruiting 81 patients to receive a PNC with either local anaesthetic or 0.9% saline. The second is the FinAPain-1 study (ISRCTN45530042), a multicentre double-blind Finnish RCT of 180 patients undergoing AKA for PAD [37]. Two catheters are placed at the time of surgery; one PNC in the perineural space and one PWC under the amputation wound. The intervention group receives ropivacaine for 72 hours whilst the control group receives 0.9% saline. Primary outcomes are pain on postoperative days 1–5, and secondary outcomes are postoperative opioid consumption, long-term PLP and CSP, and adverse events. Recruitment is expected to be complete by the end of 2017. PLACEMENT differs in that only a PNC is used, the control arm has no PNC, both AKAs and BKAs would be included, and levobupivacaine, the most commonly used local anaesthetic as identified by our systematic review, would be examined.

The PLACEMENT study will provide invaluable information on the feasibility of evaluating the role of PNCs in amputees in a full RCT. The study will facilitate a full evaluation of all aspects of the trial, including site set-up, training, recruitment and consent, logistical management, procedural technique, immediate data collection, and longer-term data capture. Qualitative interviews of patients, surgical and medical staff will identify both positive study aspects and aspects to be improved upon. Data provided from the study will clarify issues around the best primary outcome and facilitate a more accurate sample size calculation. This will provide a strong foundation upon which a future RCT can be based.

## Trial status

The study is currently in the recruitment phase.

## Additional files

Additional file 1: Spirit Checklist. (DOC 121 kb)

Additional file 2: PLACEMENT patient information sheet (DOCX 827 kb)

Additional file 3: PLACEMENT consent form (DOCX 1477 kb)

Additional file 4: Qualitative interview information sheet, healthcare professional (DOCX 1470 kb)

Additional file 5: Qualitative interview information sheets, patient (DOCX 416 kb)

Additional file 6: Qualitative interview consent form, healthcare professional (DOCX 416 kb)

Additional file 7: Qualitative interview consent form, patient (DOCX 1469 kb)

## Abbreviations

ACCEPT: Acceptance Checklist for Clinical Effectiveness Pilot Trials
AKA: Above-knee amputations
BKA: Below-knee amputations
CI: Confidence interval
CSP: Chronic stump pain
CTR: Centre for Trials Research
EQ-5D-5L: EuroQol Health Questionnaire
ISRCTN: International Standard Randomised Controlled Trial Number
NCEPOD: National Confidential Enquiry into Patient outcome and Deaths
PAD: Peripheral arterial disease
PLP: Phantom limb pain
PNC: Perineural catheter
PWC: Peripheral wound catheter
QoL: Quality of life
RCT: Randomised controlled trial
REC: Research Ethics Committee
S-LANSS: Self-completed Leeds Assessment of Neuropathic Symptoms and Signs
SPIRIT: Standard Protocol Items: Recommendations for Interventional Trials
TMG: Trial Management Group
TSC: Trial Steering Committee
UK: United Kingdom
VRS: Verbal Rating Scale
