# Supplementary material for: Perineural local anaesthetic catheter after major lower limb amputation trial (PLACEMENT): study protocol for a randomised controlled pilot study
Source: Trials. 2017 Dec 28;18:629. doi: 10.1186/s13063-017-2357-x (PMC5747086; doi:10.1186/s13063-017-2357-x)
Supplement: Supplementary file 2 — PLACEMENT patient information sheet (DOCX 827 kb) [file 13063_2017_2357_MOESM2_ESM.docx]

| 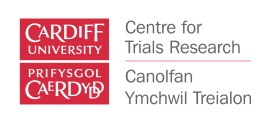 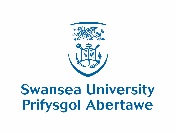 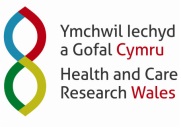 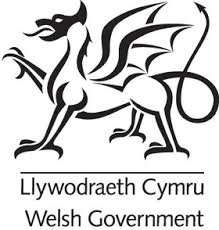  **Insert health board logo** |
| --- |


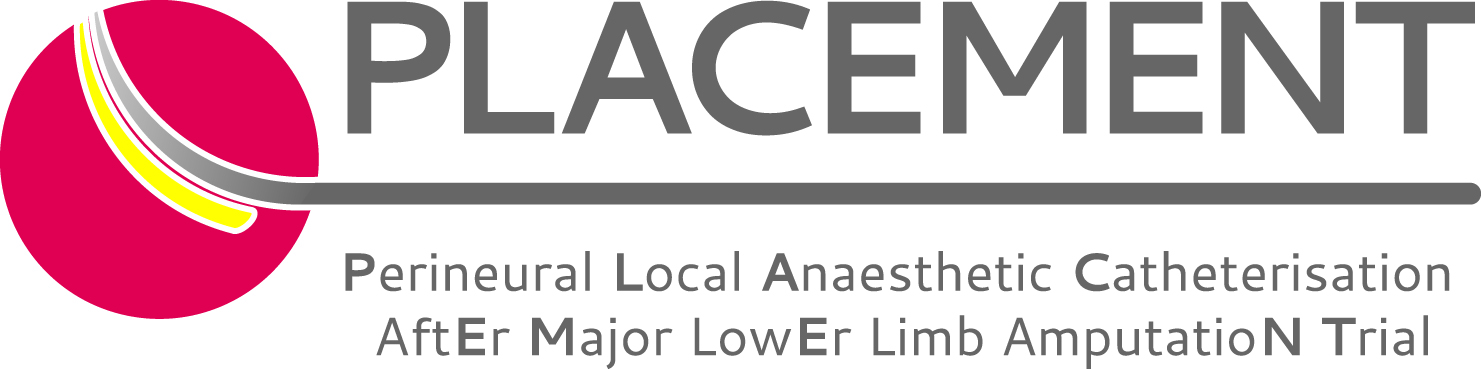


**Perineural Local Anaesthetic Catheter aftEr Major**

**lowEr limb amputatioN Trial**

Information Sheet

We are inviting you to take part in this trial run by a group of researchers at the Royal Gwent and Morriston hospitals, as well as some researchers from Cardiff and Swansea universities. Before you decide if you want to take part, we would like you to understand why the research is being done, what it would involve for you and how it will impact upon the treatment you receive after the operation.

Please read this information sheet carefully. One of our team can go through the information sheet with you and will answer any questions you have.

**Why do we want to do the trial?**

All patients undergoing an amputation get some pain after the surgery and require analgesia (pain killers) to reduce this. Controlling pain in the short term is essential to recovery immediately after the operation, especially with regards to moving out of bed with the physiotherapists. Evidence also suggests that the amount of pain an amputee experiences in the first few days after their operation can affect the amount of longer term pain and “phantom limb” they get. Good pain control is therefore essential to every patient.

We wish to look at a method of providing pain control after a patient undergoes a leg amputation.

## **What are the current treatments?**

The current “gold standard” is to provide morphine based pain killers, either by mouth or through a drip to ensure comfort after the operation. Sometimes an epidural (injection in the back) is given by the anaesthetists at the time of the operation to ease pain shortly after the procedure. This is current practice in the majority of surgical units across the UK and has been for many years. Every patient having an amputation will be reviewed by doctors and specialist nurses to ensure the correct levels of morphine and other pain killers (such as paracetamol or special medications used to reduced nerve pain) are provided.

We know that morphine provides excellent pain relief to patients with intense pain. Morphine does, however, have side effects including confusion, constipation, nausea and itching. It also interacts with many different medications. Morphine, although a good pain killer, can cause problems which lead to longer recovery time and having to spend longer in hospital.

## **What is the alternative?**

We will be trialling the use of a local anaesthetic catheter (small tube) placed at the site of the main nerve in the leg which is cut at the time of surgery. Local anaesthetic will then be given through the tube for about five days after the operation.

A local anaesthetic is a drug which has been used safely for many years in hospitals and at the dentist. It is usually injected under the skin, numbing the area. This then allows the doctor or dentist to perform minor procedures, such as removing a small mole on the skin, or taking out a tooth. It is likely that you have had a local anaesthetic before.

We wish to compare the effect of using a local anaesthetic catheter into a nerve in the leg, with the use of the usual pain killers.

**Why have I been asked to take part?**

We are inviting you to take part as you are due to have an amputation with your vascular surgeon. We aim to include a total of 50 patients in this trial from the Royal Gwent and Morriston Hospitals.

**What happens if I take part?**

If you do decide to take part in the trial you will be randomised to receive either the local anaesthetic catheter or normal care. There will be an equal chance of you receiving the catheter or receiving normal care.

The vast amount of the treatment you receive will be unchanged. If you are randomised to receive the local anaesthetic catheter, this will be inserted at the time of the operation (whilst you are under anaesthetic). The trial will not impact on what type of anaesthetic you have during the operation. Your vascular surgeon will perform the most suitable type of amputation for you and will again discuss this process with you (the trial will not influence what type of amputation you have).

As part of the amputation your surgeon will need to cut the main nerve in your leg. When he or she is doing this they will be able to place the catheter alongside the nerve inside the leg. The catheter tube will be brought out through the skin at a point away from the wound. The catheter will remain in place with an infusion (constant slow stream) of a local anaesthetic for the first five days after the amputation. The catheter will normally be removed at the end of day five after your amputation. The removal is a painless process which can be performed on the ward by the nurse looking after you.

**During your hospital stay**

Your surgeon and anaesthetist will manage your care in the same way as all patients undergoing an amputation prior to the procedure; by taking part in the trial we will not need to perform any additional tests. We will however ask you about your current level of pain and what you are taking to help relieve this, and write down some basic information about you, your medical history and your current medications. You will also be asked to complete some questionnaires.

When the surgeon has nearly completed the amputation he or she will be ready to cut the major nerve in your leg. At this point you will be randomly assigned to either receive the nerve catheter or not. If you are selected to have the catheter placed, your surgeon will insert the catheter tube alongside the nerve and bring this out of the skin away from the wound.

After the surgery you will receive the normal care provided to all patients undergoing an amputation. When back on the ward the nursing staff will keep a close eye on your observations and will ask you to rate your levels of pain and record this (this is normal practice for all post-operative patients). You will receive as much morphine and other types of pain killers as you need to remain comfortable. This would all be recorded by the nurses as routine, but as part of the trial we will use this information to see whether the local anaesthetic treatment is effective. A member of the research team will come to see you every day for the first 5 days after the operation and will ask you some additional questions about how well your pain is controlled and whether you are suffering from side effects associated with morphine.

If needed, further pain relief will be provided (in addition to the local anaesthetic catheter if you have one). This can be given as normal (i.e. the same as if you did not have a local anaesthetic catheter) either by mouth or through a drip. The catheter does not limit the amount of morphine that can be given to control pain.

**5 days after the operation**

Five days after the operation the catheter will be removed and your recovery and time spent in hospital will continue as normal (such as time spent with physiotherapists, occupational therapists and social workers to ensure you are ready to return home, and your home is well set up for your new circumstances). Information about your stay in hospital will also be collected by the research team, again to see if the new treatment had any effect on this.

**At 6 week and 6 month follow-up appointments.**

In addition to the normal follow up you would receive from the hospital 6 weeks after your operation, if you choose to be involved in the trial we will contact you or your preferred alternative contact person, normally via telephone, around 6 months after your amputation. At both your usual 6 week follow-up appointment and at 6 months, the research team will ask about any symptoms of pain you may be experiencing in the long term and go through some questionnaires. We would want to know about any pain medications you were taking at that point (we will also ask your GP so it is not essential for you to remember). This should take around half an hour of your time. We would also like to look at your medical records so that we can collect information on healthcare costs such as prescriptions, visits to the GP and so on.

**Longer term follow up using health data**

After the trial, we would like to see how you are doing in the longer term by using routinely collected health data. This is information that your GP and hospital collect when looking after you. We will look at this information to explore the long term effects of the nerve catheter versus normal care.

The reason we want to look at this information is to assess the wider effects on your well-being and health following the trial. The research team will provide details such as name, NHS number, date of birth, postcode and gender to identify you to NHS Digital in order for those professionals to provide relevant information about your ongoing health. NHS Digital will provide information on how trial participants have used different health services (e.g. how many hospital visits) and for what reasons.

NHS digital will provide data to Cardiff University. No identifiable data will be sent to the database. Instead, a study number will be assigned to each individual and this will be used to join pieces of information together. Data viewed by the research team will not be identifiable. In other words, when we look at your health information, all we will see is a database containing numbers. A Data Manager will use the study number to identify a person and join up the information. We will not know who is who in the database. The data will be held on a secure server and only the research team will have access to it.

**Qualitative interview study**

A few patients in the trial will be asked by a member of the research team to discuss the experience of taking part in the trial and about their health. If you agree, a researcher will give you some information about the additional study and what will be required. After you have had time to go through this and ask any questions you might have, you will be asked if you are happy to take part. If so you will be asked to sign a consent form to confirm that you understand this part of the study and agree to participate. If you do not take part this will not affect your involvement in the research or ongoing health care.

Do I have to take part?

No. Participation in the trial is entirely voluntary and you are free to refuse to take part or withdraw from the trial at any time without having to give a reason. If you decide not to take part or to withdraw from the trial, this will not affect the care that you receive. You would receive the normal pain relief that all amputees are currently provided with after their operation.

Can I choose which part of the trial to be in?

No. To get the most reliable information about how well the local anaesthetic treatment works we must ensure that each patient is given an equal chance of being given the nerve catheter or not, and that this is chosen at random.

**What are the possible advantages of taking part?**

By participating in this trial there may be a possibility that if the treatment works, your pain will be reduced, leading to a reduction in morphine use and its side effects, which could improve your recovery. You will receive a gift voucher at the follow-up consultations to say thank you for taking part, and any travel expenses will be reimbursed.

In addition, you will be helping us to answer questions about the treatment of leg amputation that should result in better care for patients needing this in the future.

**What are the possible disadvantages and risks of taking part?**

Taking part in the trial will mean that we ask you to give up some of your time during your hospital stay and at your follow-up appointments.

As with any drug there are possible side effects, however side effects of local anaesthetics are rare and the majority of patients do not have any problems. Allergies to local anaesthetics can occur but are rare, occurring in less than one in every thousand patients. Allergies can cause a reaction in the skin injected, causing redness and swelling, or in very rare cases more severe reactions. Receiving too much local anaesthetic can cause problems to the heart which trigger a high heart rate and low blood pressure. This happens mainly when a large amount of local anaesthetic is injected all at the same time, and can be avoided by giving a slow infusion and ensuring the dose is based on how much the patient weighs.

From our experience and from reports in other hospitals across the world, there have been very few reported problems with local anaesthetic catheters. Although uncommon, there is a chance that you may get an infection caused by the catheter. This can cause cellulitis (redness and heat in the top layer of the skin) around the catheter. This can be treated by giving antibiotics either by mouth or through a drip.

**Is there an impact on possible pregnancy and breast feeding?**

There is some evidence from animal studies that local anaesthetics can be harmful to the baby if given in early pregnancy. Therefore if you are a woman of child bearing age, the trial team will need to do a pregnancy test before we can accept you in the trial. You will also be asked to either refrain from sexual intercourse or use effective contraception for the week following the operation, so that any local anaesthetic given via the nerve catheter is out of your system before there is any chance of you becoming pregnant.

Although a tiny amount of local anaesthetic does get into breast milk, the amount is very small and not thought to be harmful, so if you are breastfeeding you may continue to do so while participating in the trial.

**Will my records, and the fact that I am taking part be kept confidential?**

Yes, all information collected about you during the course of the trial will be kept strictly confidential. Your GP and other doctors involved in your care will be informed about your participation in the trial, and will be kept up to date with your progress to ensure your safety, but they will not see any answers to trial questionnaires.

Your medical records may be examined by the research team but will be kept confidential. Your name (or any other identifiable information) will not be given out or appear on any publications**.** We will store the information collected during this research trial (including your details) for a minimum of 15 years in accordance with clinical trial regulations. The research team follow ethical and legal practice and all trial information will be stored securely and confidentially by the research team at Cardiff University in accordance with the 1998 Data Protection Act. Only the study team will have access to the information.

Other than the researchers involved in your trial, it may be required for certain authorities to look at the data we collect. This is to ensure that we are running the trial in accordance with the relevant regulations to ensure that all patients and data are being treated correctly.

**What if I do not want to carry on being part of the trial?**

You can decide to stop taking part in the trial at any time and without needing to give a reason. If you wish to withdraw, you can contact the trial manager or a member of the research team, or let them know next time they contact you. In order for us to understand the reasons why patients withdraw from the trial, we may ask you why you have decided to withdraw. However, you do not have to give any reasons.

It is usual practice to keep the information we have collected to help draw conclusions about the treatment you received. If however you do not want your information used you may request that it be withdrawn from the study as well.

If you decide to stop taking part, your current and future medical care and your legal rights will not be affected in any way.

**What will happen to the results of the research?**

We hope that the results of the trial will improve the care of many patients in the future. At the end of the trial, the results will be published in medical journals and presented at medical conferences. This will allow us to tell other doctors and healthcare professionals across the world about the results of the trial.

Information that identifies you **will not** be presented. We can provide you with information of where to access the published trial data. A copy of the results can be sent to you if you would like.

**Who has funded and approved the study?**

The trial is funded by money from the Welsh Government through the Health and Care Research Wales, Research for Patient and Public Benefit (RfPPB) scheme.

The trial has been approved by an independent NHS Research Ethics Committee (REC) 16/WA/0353. The committee makes sure that the study is conducted ethically to protect the safety, rights, wellbeing and dignity of the patient in line with the requirements of Clinical Trial Regulations.

**What if there is a problem?**

If you experience any side effects or complications as a result of taking part in the trial there are no compensation arrangements over and above the usual routes available to any patient treated within the NHS. If at any point you are unhappy with any aspect of the trial, please advise your Surgeon or the research team at the Centre for Trials Research, Cardiff University (contact numbers below). If you remain unhappy and wish to formally complain, you can do this through the normal NHS complaints procedure. Taking part in the trial will not affect your legal rights.

**What do I need to do now?**

If you agree to take part in the trial please tell your Surgeon that you are happy to take part. You may want to discuss the trial with a member of the research team. Once you are happy with the requirements of taking part you will be asked to sign a consent form to confirm you are happy to participate in the trial.

Thank you for reading this information sheet and considering participation in this trial. Our team is experienced and dedicated to doing this important trial to the highest international standards, and helping to improve the future care of patients.

**Should you have any further questions or require further information about taking part you can contact (during normal working hours):**

PLACEMENT Trial Manager

South East Wales Trials Unit, Centre for Trials Research, Cardiff University, Heath Park, Cardiff, CF14 4YS

[Tel: 02920](Tel:02920) 687609

Email: PLACEMENT-TRIAL@cardiff.ac.uk

Please note that this number is only for queries regarding the trial; if you have an urgent medical problem please contact your surgeon/doctor in the normal way.

**The Principal Investigator for this site is:-**

Insert PI details
