# Supplementary material for: Perineural local anaesthetic catheter after major lower limb amputation trial (PLACEMENT): study protocol for a randomised controlled pilot study
Source: Trials. 2017 Dec 28;18:629. doi: 10.1186/s13063-017-2357-x (PMC5747086; doi:10.1186/s13063-017-2357-x)
Supplement: Supplementary file 3 — PLACEMENT consent form (DOCX 1477 kb) [file 13063_2017_2357_MOESM3_ESM.docx]

**CONSENT FORM**

(Please **initial** each box and sign in full at the bottom of the page)

| 1. | I confirm that I have read and understood the Information Sheet (version 1.2, dated 08/05/2017) for the PLACEMENT trial. I have had the opportunity to consider the information, ask questions and have had these answered satisfactorily. |  |
| --- | --- | --- |
| 2. | I understand that my participation is voluntary and that I am free to withdraw at any time, without giving any reason and without my usual medical care or legal rights being affected. |  |
| 3. | I understand the trial is randomised and no one can pick which treatment I receive. I understand that I will be randomised to receive either a nerve catheter or usual care. If I am allocated to the treatment arm of the trial I will have a nerve catheter placed at the time of my amputation. I consent to the insertion of the nerve catheter at the time of my amputation. |  |
| 4. | I agree for my GP to be informed of my participation in this trial. I understand that the research team may be required to look in my medical notes or contact my GP to ask about my health and medications. I give permission for them to do this. |  |
| 5. | I understand that a member of the research team will need to contact me by telephone to complete a survey about my health. I give permission for them to do so. |  |
| 6. | I understand that if a member of the research team cannot reach me directly they may need to speak to an alternative contact person on my behalf. I give permission for them to do so. |  |
| 7. | I understand that a member of the research team may need to contact me to carry out an interview about my health and experiences in the trial (Qualitative Interviews). I give permission for them to do so. |  |
| 8. | I understand that information collected about me that is held and maintained by NHS Digital and other central UK NHS bodies, may be collected from my medical records and other health-related records and looked at by the research team and responsible practitioners during the trial. I give permission for these individuals to have access to these records and for them to be used in this research on the understanding that all information will remain confidential. |  |
| 9. | I understand that information collected about me (including name and address) will be held at the Centre for Trials Research, Cardiff University according to the 1998 Data Protection Act. I understand that this information will be kept strictly confidential and that no personal information will be used in the study report or publications. |  |
| 10. | I agree to regulatory authorities accessing the data obtained in this study where it is relevant to my taking part in research, on the understanding that all data will remain confidential. |  |
| 11. | I agree to take part in the above trial. |  |

Name of Participant Signature Date

Name of Person taking consent Signature Date

**When completed, store White copy in Site File; Green for Medical Notes; Yellow for participant**

**Please fax a copy to CTR: 02030095402**
