# Supplementary material for: Perineural local anaesthetic catheter after major lower limb amputation trial (PLACEMENT): study protocol for a randomised controlled pilot study
Source: Trials. 2017 Dec 28;18:629. doi: 10.1186/s13063-017-2357-x (PMC5747086; doi:10.1186/s13063-017-2357-x)
Supplement: Supplementary file 4 — Qualitative interview information sheet, healthcare professional (DOCX 1470 kb) [file 13063_2017_2357_MOESM4_ESM.docx]

**CONSENT FORM FOR QUALITATIVE STUDY (HCP)**

(Please **initial** each box and sign in full at the bottom of the page)

| 1. | I confirm that I have read and understood the Information Sheet for Qualitative Study: Health Care Professional (version V1.0, dated 24.10.2016) for the PLACEMENT Qualitative Study. I have had the opportunity to consider the information, ask questions and have had these answered satisfactorily. |  |
| --- | --- | --- |
| 2. | I understand that my participation is voluntary and that I am free to withdraw at any time, without giving any reason, without my legal rights being affected. |  |
|  |  |  |
| 3. | I am willing to talk with a member of the research team about my experiences and views on the care of patients undergoing major limb amputation and about being involved in the trial. I give permission for my interview with the researcher to be audio-recorded. I understand that information collected about me during the interview will be treated with the strictest confidentiality and may be listened to by the research team but by no-one else. The recording will not be labelled with my name and any written record or report derived from it will be fully anonymised. I understand that what I say in the interview may be quoted word for word but the quote will be anonymous. |  |
| 4. | I am willing to participate in a group interview (focus group) to share my experiences and opinions about the care of adults who have undergone a leg amputation with researchers and other participants in the group. I give permission for the focus group to be audio-recorded. I understand that what I say in the focus group may be quoted word for word but the quote will be anonymous. |  |
| 5. | I understand that information collected about me (including name and address) will be held at the Centre for Trials Research, Cardiff University according to the 1998 Data Protection Act. I understand that this information will be kept strictly confidential and that no personal information will be used in the study report or publications. |  |
| 6. | I agree to regulatory authorities accessing the data obtained in this study where it is relevant to my taking part in research, on the understanding that all data will remain confidential. |  |
| 7. | I agree to take part in the above Qualitative Study. |  |

Name of Participant Signature Date

Name of Person taking consent Signature Date

**When completed, store White copy in Site File; Yellow for participant**

**Please fax a copy to CTR: 02030095402**
