# Supplementary material for: Perineural local anaesthetic catheter after major lower limb amputation trial (PLACEMENT): study protocol for a randomised controlled pilot study
Source: Trials. 2017 Dec 28;18:629. doi: 10.1186/s13063-017-2357-x (PMC5747086; doi:10.1186/s13063-017-2357-x)
Supplement: Supplementary file 5 — Qualitative interview information sheets, patient (DOCX 416 kb) [file 13063_2017_2357_MOESM5_ESM.docx]

| 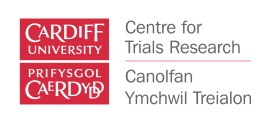 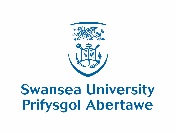 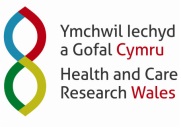 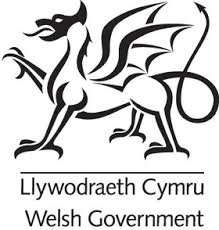  **Insert health board logo** |
| --- |


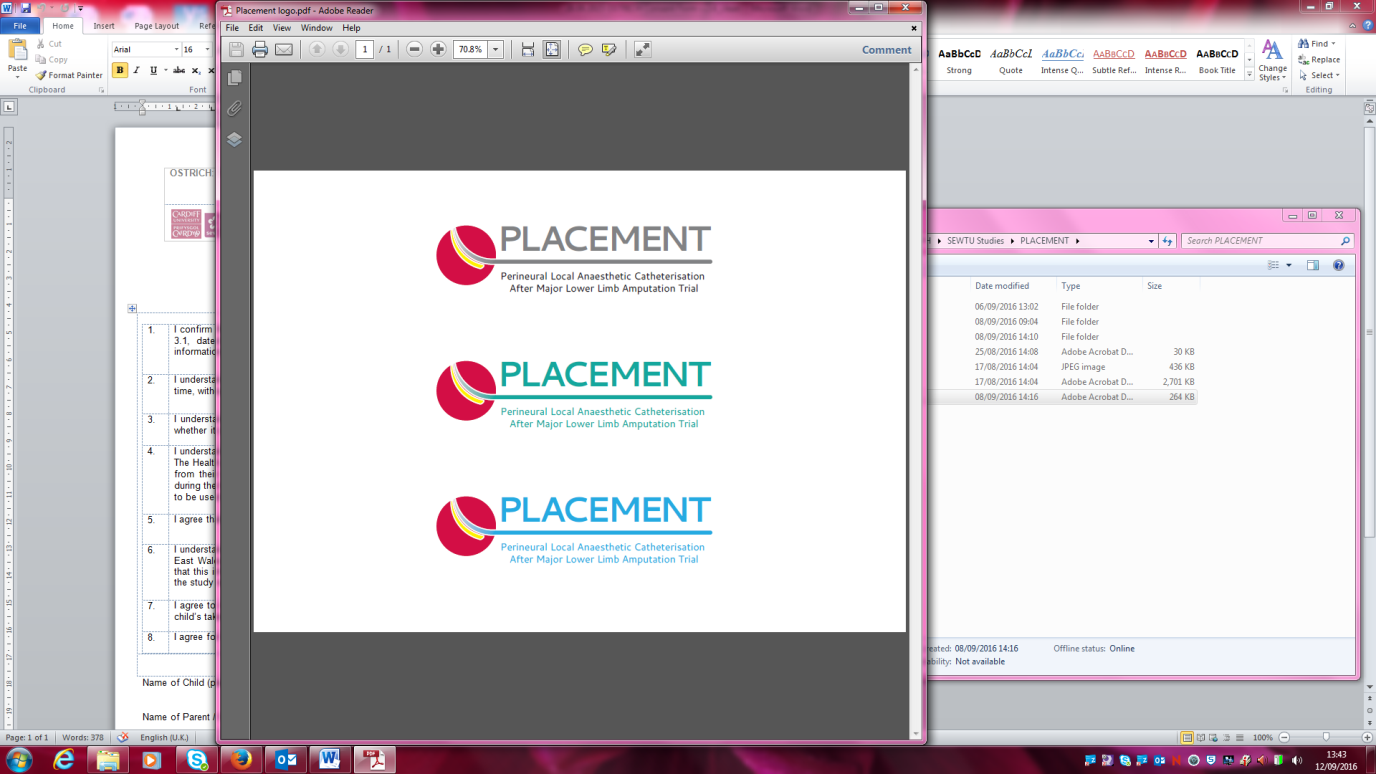


**Perineural Local Anaesthetic Catheter aftEr Major**

**lowEr limb amputatioN Trial**

Information Sheet for Qualitative Study: Patient

You are taking part in a trial called PLACEMENT run by a group of researchers at the Royal Gwent and Morriston hospitals, and researchers from Cardiff and Swansea universities. The trial looks at pain control after a patient undergoes a leg amputation. It compares the effect of using a local anaesthetic catheter into a nerve in the leg, with the use of the usual pain killers.

We are asking if you would like to take part in an additional interview in relation to the PLACEMENT trial. During the interview, a researcher will ask you about your personal experience following amputation, your views on pain treatment, and how it feels to be part of a research trial. We may also ask you to take part in a group interview (focus group) and share your opinions about the care of adults who have undergone a leg amputation with other participants in the group (other patients and health care professionals).

Please read this information sheet carefully. One of our team can go through the information sheet with you and can answer any questions you have.

**Why have I been asked to take part in the Qualitative Study?**

We are inviting you to take part as you are already taking part in the PLACEMENT trial. We would like to hear about your experiences following amputation, and your views on pain treatment in your own words.

**What happens if I take part?**

If you agree, you will be asked to sign a consent form to confirm that you understand the qualitative study and agree to participate. A researcher will contact you to discuss the best time to speak to you. If you agree, we would like to talk to you on two separate occasions. Firstly, between 4 days and 4 weeks after your operation: this is likely to be during your hospital visit. If this is not possible, we would like to talk to you when possible at a time and location convenient to you (' this can be in your home if it is convenient). Secondly, about 6-9 months after your operation at a location convenient to you ( this can be in your home if it is convenient), to find out how your experience following the amputation and pain management has changed over time. You do not have to take part in a second interview if you do not wish to. The length of the interview may vary, and any time you can give will be helpful, but we expect it to take about 45-60 minutes. In order to help us with our research we would like to audio-record our conversation.

Core Outcomes study

We **may** also ask you to take part in a focus group at a time and place that is convenient for you, to discuss general issues important to adults who have undergone leg amputation. The focus group will last around 60-90 minutes. In order to help us with our research we would like to audio-record the focus group. This part of the study is designed to develop of a list of the most important outcomes following amputation (Core Outcome Sets: COS), which will tell us what should be reported on in all studies of amputation research. If you agree, following the interviews and/or focus groups, we will produce a list of core outcomes and will contact you again to ask which you think are most and least important on the list. We will do this by asking you to complete a series of surveys (this could be online, on paper, or verbally).

**Ethical Approval**

The study has been approved by the NHS Research Ethics Committee (REC) 16/WA/0353. The committee makes sure that the study is conducted ethically and in accordance with the requirements of the Clinical Trials Regulations.

Do I have to take part?

No. Your participation is entirely voluntary and you are free to refuse to take part or withdraw at any time without having to give a reason. If you decide not to take part or to withdraw, this will not affect the care that you receive.

**What are the possible disadvantages and advantages of taking part?**

Participation in the interview/focus group is not likely to involve any particular risks although participating in such a discussion may bring back memories of a difficult and distressing time in your life. If you do become upset we can stop the interview at any time and the researcher can direct you to more support if required. The main disadvantage in taking part is giving up your time to talk to the researcher. The main advantage is that you will help improve understanding of the experiences of people who have undergone an amputation.

**Will my taking part be kept confidential?**

Yes. All the information, including any personal information (e.g. your name), will be kept completely confidential. Recordings will not be labelled with your name. Any computer file containing a record of your conversation, and any written report of the research, will have your name removed. Written quotes of what you say in the interviews/focus group may be used word for word, but quotes will be anonymised. All data held by the researchers will be stored to meet the requirements of the Data Protection Act and study related records will be stored for a minimum of 15 years. Other than the researchers involved in the study, it may be required for certain authorities to look at the data we collect. This is to ensure that we are running the study properly and that all patients and data are being treated correctly.

**What if I do not want to carry on being part of the study?**

You can decide to stop taking part in the trial and/or the qualitative study at any time, without needing to give a reason. If you wish, you can contact the trial manager or a member of the research team, or let them know next time they contact you. In order for us to understand the reasons why patients withdraw from research, we may ask you why you have decided to withdraw. However, you do not have to give any reasons.

It is usual practice to keep the information we have collected to help draw conclusions about the treatment you received. If however you do not want your information used you may request that it be withdrawn from the trial and qualitative study as well.

If you decide to stop taking part, your medical care and your legal rights will not be affected in any way.

**What will happen to the results of the research study?**

The results are likely to be published in medical journals over the next few years. You will not be personally identified in any report or publication.

**Who has funded and approved the study?**

The study is funded by money from the Welsh Government through the Research for Patient and Public Benefit (RfPPB) scheme.

It has been approved by an independent NHS Research Ethics Committee. Their job is to protect your safety, rights, wellbeing and dignity.

**What if there is a problem?**

If at any point you are unhappy with any aspect of the qualitative study, please advise the research team at the Centre for Trials Research, Cardiff University (contact numbers below). If you remain unhappy and wish to formally complain, you can do this through the normal NHS complaints procedure. Taking part in the qualitative study and trial will not affect your legal rights.

**What do I need to do now?**

You may want to discuss the study with a member of the research team. If you agree to take part in the qualitative study, you will be asked to sign a consent form to confirm that you understand the study and agree to participate. A member of the research team will then contact you to arrange an interview (usually between 4 days and 4 weeks after your operation) and again at a later point (approximately 6-9 months after).

Thank you for reading this information sheet and considering participation in this study. Our team is experienced and dedicated to doing this important qualitative study to the highest international standards, and helping to improve the future care of patients.

**Should you have any further questions or require further information about taking part you can contact (during normal working hours):**

PLACEMENT Trial Manager

South East Wales Trials Unit, Centre for Trials Research, Cardiff University, Heath Park, Cardiff, CF14 4YS

[Tel: 02920](Tel:02920) 687609

Email: PLACEMENT-Trial@Cardiff.ac.uk

Please note that this number is only for queries regarding the trial; if you have an urgent medical problem please contact your surgeon/doctor in the normal way.

**The Principal Investigator for this site is:-**

Insert PI details
