# Supplementary material for: Perineural local anaesthetic catheter after major lower limb amputation trial (PLACEMENT): study protocol for a randomised controlled pilot study
Source: Trials. 2017 Dec 28;18:629. doi: 10.1186/s13063-017-2357-x (PMC5747086; doi:10.1186/s13063-017-2357-x)
Supplement: Supplementary file 6 — Qualitative interview consent form, healthcare professional (DOCX 416 kb) [file 13063_2017_2357_MOESM6_ESM.docx]

| 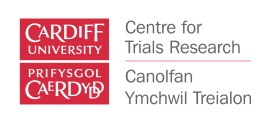 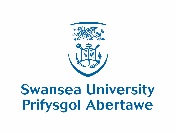 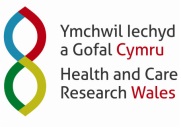 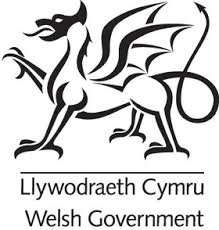  **Insert health board logo** |
| --- |


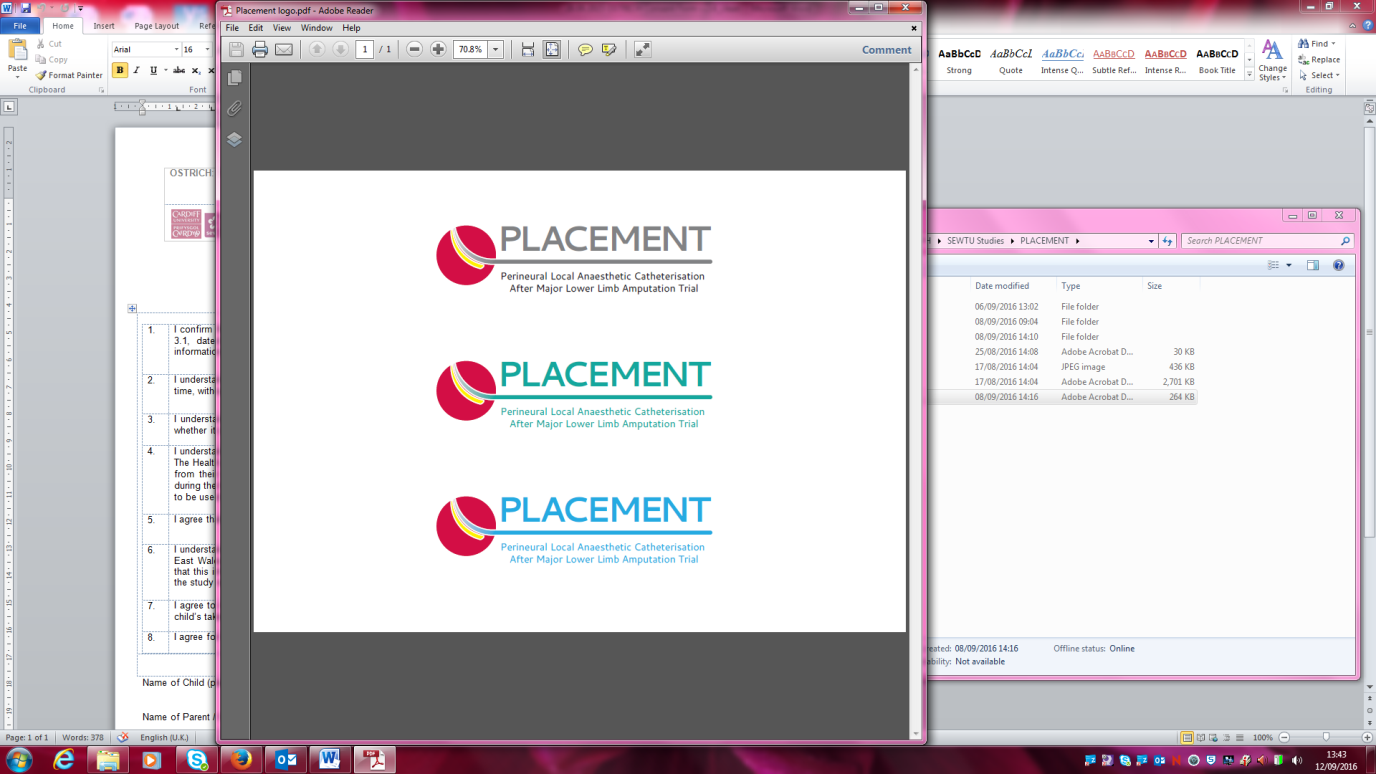


**Perineural Local Anaesthetic Catheter aftEr Major**

**lowEr limb amputatioN Trial**

Information Sheet for Qualitative Study:

Health Care Professional

We would like to invite you to take part in an interview as part of the PLACEMENT trial. We may also ask you to take part in a group interview (focus group) and share your opinions about the care of adults who have undergone a leg amputation with other participants in the group (patients and other health care professionals). Please read this information sheet carefully and consider whether or not you would like to take part. Please ask us if anything is unclear or if you would like more information.

**What is the purpose of the study?**

The study aims to explore the views of patients and health care professionals on what is most important in the care and management of patients undergoing major limb amputation. We will also identify important outcomes (Core Outcome Sets: COS) which will tell us what should be measured and reported on in all studies of amputation research.

**Why have I been asked to take part in the Qualitative Study?**

We are approaching patients who have undergone major limb amputation and health care professionals who are involved in the care of patients who have undergone amputation.

**What happens if I take part?**

If you agree, you will be asked to sign a consent form to confirm that you understand the study and agree to participate. A researcher will contact you to discuss the best time to speak to you in an interview and/or focus group at a time that is convenient for you. Interviews can be conducted over the telephone or face-to-face and will last 20-60 minutes.

If you participate in a focus group it will last around 60-90 minutes. In order to help us with our research we would like to audio-record the discussions. After we have spoken to patients and health care professionals we will produce a list of core outcomes and we will contact you again to ask which you think is most and least important on the list. We will do this by asking you to complete a survey (this could be online, on paper, or verbally).

**Ethical Approval**

The study has been approved by the NHS Research Ethics Committee (REC) 16/WA/0353. The committee makes sure that the study is conducted ethically and in accordance with the requirements of the Clinical Trials Regulations.

Do I have to take part?

No. Your participation is entirely voluntary and you are free to refuse to take part or withdraw at any time without having to give a reason. The care that patients receive will not be affected if they decide not to take part or to withdraw.

**What are the possible disadvantages and advantages of taking part?**

The main disadvantage in taking part is giving up your time to talk to the researcher. The main advantage is that you will have the opportunity to help improve understanding of the experiences of people who have undergone an amputation.

**Will my taking part be kept confidential?**

Yes. All the information, including any personal information (e.g. your name), will be kept completely confidential. Recordings will not be labelled with your name. Any computer file containing a record of your conversation, and any written report of the research, will have your name removed. Written quotes of what you say in the interview/focus group may be used word for word, but quotes will be anonymised. All data held by the researchers will be stored to meet the requirements of the Data Protection Act and study records will be stored for a minimum of 15 years. Other than the researchers involved in the study, it may be required for certain authorities to look at the data we collect. This is to ensure that we are running the study properly and that all patients and data are being treated correctly.

**What if I do not want to carry on being part of the study?**

You can decide to stop taking part in the qualitative study at any time and without needing to give a reason. If you wish, you can contact the trial manager or a member of the research team, or let them know next time they contact you. In order for us to understand the reasons why participants withdraw, we may ask you why you have decided to withdraw. However, you do not have to give any reasons. It is usual practice to keep the information we have collected. If however you do not want your information used you may request that it be withdrawn.

**What will happen to the results of the research study?**

The results are likely to be published in medical journals over the next few years. You will not be personally identified in any report or publication.

**Who has funded and approved the study?**

The study is funded by money from the Welsh Government through the Research for Patient and Public Benefit (RfPPB) scheme. It has been approved by an independent NHS Research Ethics Committee. Their job is to protect your safety, rights, wellbeing and dignity.

**What if there is a problem?**

If at any point you are unhappy with any aspect of the qualitative study, please advise the research team at the Centre for Trials Research, Cardiff University (contact numbers below). If you remain unhappy and wish to formally complain, you can do this through the normal NHS complaints procedure. Taking part in the qualitative study and trial will not affect your legal rights.

**What do I need to do now?**

If you agree to take part in the qualitative study, you will be asked to sign a consent form to confirm that you understand the study and agree to participate. We will contact you to arrange a suitable time for interview and/or focus group. If you have any questions or would like to inform us that you would like to take part, please contact a member of the study team using the contacts provided below.

Thank you for reading this information sheet and considering participation in this study. Our team is experienced and dedicated to doing this important trial and qualitative study to the highest international standards, and helping to improve the future care of patients.

**Should you have any further questions or require further information about taking part you can contact (during normal working hours):**

PLACEMENT Trial Manager

South East Wales Trials Unit, Centre for Trials Research, Cardiff University, Heath Park, Cardiff, CF14 4YS

[Tel: 02920](Tel:02920) 687609

Email: PLACEMENT@cardiff.ac.uk

**The Principal Investigator for this site is:-**

Mr/Mrs/Dr XXXXXXXX

XXXXXXXXXXXXXXX
